# Biofilms for Turbidity Mitigation in Oil Sands End Pit Lakes

**DOI:** 10.3390/microorganisms9071443

**Published:** 2021-07-04

**Authors:** Heidi L. Cossey, Mian Nabeel Anwar, Petr V. Kuznetsov, Ania C. Ulrich

**Affiliations:** Department of Civil & Environmental Engineering, University of Alberta, Edmonton, AB T6G 1H9, Canada; cossey@ualberta.ca (H.L.C.); miannabe@ualberta.ca (M.N.A.); pkuznets@ualberta.ca (P.V.K.)

**Keywords:** biofilm, biostabilization, turbidity, end pit lake, Base Mine Lake, oil sands, tailings

## Abstract

End pit lakes (EPLs) have been proposed as a method of reclaiming oil sands fluid fine tailings (FFT), which consist primarily of process-affected water and clay- and silt-sized particles. Base Mine Lake (BML) is the first full-scale demonstration EPL and contains thick deposits of FFT capped with water. Because of the fine-grained nature of FFT, turbidity generation and mitigation in BML are issues that may be detrimental to the development of an aquatic ecosystem in the water cap. Laboratory mixing experiments were conducted to investigate the effect of mudline biofilms made up of microbial communities indigenous to FFT on mitigating turbidity in EPLs. Four mixing speeds were tested (80, 120, 160, and 200 rpm), all of which are above the threshold velocity required to initiate erosion of FFT in BML. These mixing speeds were selected to evaluate (i) the effectiveness of biofilms in mitigating turbidity and (ii) the mixing speed required to ‘break’ the biofilms. The impact of biofilm age (10 weeks versus 20 weeks old) on turbidity mitigation was also evaluated. Diverse microbial communities in the biofilms included photoautotrophs, namely cyanobacteria and Chlorophyta (green algae), as well as a number of heterotrophs such as *Gammaproteobacteria, Desulfobulbia, and Anaerolineae*. Biofilms reduced surface water turbidity by up to 99%, depending on the biofilm age and mixing speed. Lifting and layering in the older biofilms resulted in weaker attachment to the FFT; as such, younger biofilms performed better than older biofilms. However, older biofilms still reduced turbidity by 69% to 95%, depending on the mixing speed. These results indicate that biostabilization is a promising mechanism for turbidity mitigation in EPLs.

## 1. Introduction

Over 1.3 billion m^3^ of oil sands tailings are currently being stored in large above-ground impoundments, referred to as tailings ponds, in northern Alberta [1]. All of these tailings must be reclaimed and integrated into the surrounding landscape after oil sands mines are closed. Tailings are generally made up of solids (sand, silt, and clay), residual bitumen, and oil sands process-affected water (OSPW) that typically contains elevated levels of salts and naphthenic acids (NAs) [2]. Fine-grained tailings, referred to as fluid fine tailings (FFT), have solids that consist primarily of clay (less than 2 µm) and silt (less than 44 µm) particles. One proposed method of oil sands tailings reclamation is end pit lakes (EPLs), which are engineered water bodies comprised of thick FFT deposits (10–80 m) stored beneath a water cap (3–10 m) in decommissioned open pits. EPLs, which are commonly referred to as pit lakes in other mining industries, are permanent features of most open pit mines both in Canada and worldwide [3]. However, the design and configuration of pit lakes and the type of tailings stored in pit lakes vary with different industries and operators. Due to the fine-grained nature of FFT and the slow consolidation rate, there are unique challenges associated with reclaiming oil sands tailings in EPLs [4].

The design of EPLs allows for tailings to naturally dewater (consolidate) over time, while the water cap (hereinafter referred to as surface water) provides habitat for an aquatic ecosystem [5]. However, settlement and resuspension of FFT particles (especially clay particles) in the surface water generates turbidity, which may limit light penetration and thereby be detrimental to the development of a healthy aquatic ecosystem in an EPL [5]. Furthermore, turbidity can result in lower dissolved oxygen concentrations in EPL surface water, which would negatively impact the biodegradation of acutely toxic NAs that are present in OSPW [6,7,8].

Base Mine Lake (BML), operated by Syncrude Canada Ltd. (Syncrude), is the first full-scale demonstration EPL for oil sands tailings. It was initially developed in 2012 and currently consists of roughly 45 m of FFT and 9 m of surface water. Because BML is the first of its kind, it is continually monitored and studied in order to determine the viability of this proposed tailings reclamation method. Tedford et al. [9] measured seasonal turbidity changes in BML’s surface water over a period of three years and reported turbidity as high as 308 NTU (nephelometric turbidity units) at a depth of 2.5 m after lake turnover in fall 2015. Research has shown that even a relatively small increase in turbidity, from 10 to 50 NTU, can have a rapid and negative impact on aquatic metabolism [10]. Turbidity in BML is thought to be due to the settling of fine particles suspended in the surface water and the resuspension of fine particles from the FFT–water interface [11]. There are a number of processes that could conceivably contribute to particle suspension and turbidity in BML, including, but not limited to, wind waves and other wind-driven processes, convection, gas ebullition, and pore water expression [11]. In 2016, BML was dosed with a chemical coagulant, alum, which substantially reduced the surface water turbidity that year [12]. However, once the aluminum ions are consumed down to background levels, this coagulation effect may disappear [13]. The long-term trends in turbidity following coagulant addition in BML are unclear, though turbidity measurements in 2017 and 2018 suggest turbidity may be returning to pre-alum levels [12]. As such, biological mechanisms have been proposed as a new approach to turbidity reduction in BML.

Biofilms formed on the EPL mudline (the FFT–water interface) have been proposed as a novel solution for mitigating turbidity in the long term. Biofilms have been found to have a stabilization effect, referred to as biostabilization, whereby they reduce the ability of sediments to resuspend and of contaminants to re-mobilize from a sediment–water interface in comparison to purely mineral sediments [14,15]. Biostabilization has been shown to significantly increase the energy required to erode sediments, thereby reducing turbidity in the overlying water [15,16]. These effects are thought to be due to the secretion of gel-like extracellular polymeric substances (EPS), made up of lipids, polysaccharides, proteins, and nucleic acids, which attach microbial cells to particle surfaces [14,16,17,18,19,20]. EPS are hydrated, which causes them to swell on exudation and fill pore spaces [19,21]. This facilitates stabilization and results in EPS making up a large volume of the biofilm matrix. Stabilizing biofilms often consist of multiple layers of multispecies communities, which may include diatoms, green algae, photoautotrophic cyanobacteria, and other phototrophic and heterotrophic bacteria [18,19,20,22]. Though consolidation and electrochemical interactions are thought to also affect the stabilization of bed sediments [23,24], there is a consensus that the secretion of EPS by microorganisms is the primary reason for the stabilization effects [14,19,25,26,27].

Previous work has largely focused on biofilm formation and stabilization of marine [21,26,28,29] and fresh water [16,19] sediments. However, the high surface area to volume ratio and the charged nature of clay particles in FFT should be advantageous for microbial growth and biostabilization [20]. Bordenave et al. [30] observed increased clay aggregation and sedimentation in Albian sands tailings under nitrate-reducing and methanogenic conditions and attributed this to the development of microbial biofilms on fine particles. Furthermore, Reid et al. [14] compared biostabilization in fresh and aged FFT in tailings ponds and found that aged tailings had greater microbial diversity and biofilms extending past the FFT–water interface, both of which led to higher biostabilization in aged tailings. Conversely, fresh tailings had lower microbial diversity, and only a shallow biofilm formed at the FFT–water interface [14]. This current study uses laboratory mixing tests to evaluate biostabilization as a possible mechanism for turbidity reduction in BML. This work is the first to investigate EPL turbidity mitigation using mudline biofilms made up of microbial communities indigenous to FFT. The results of this study will help mitigate turbidity issues that may be detrimental to the long-term success of EPLs.

## 2. Materials and Methods

### 2.1. Sample Collection and Characterization

#### 2.1.1. BML Surface Water and FFT Collection

Grab samples of BML FFT and BML surface water were collected in 20 -liter pails by Syncrude in August 2017 and stored at the University of Alberta at 4 °C until use. BML FFT was collected at a depth of 10.2 m below the lake surface (approximately 1.0 m below the FFT–water interface). This depth was chosen because particles near the FFT–water interface are fine-grained and are most likely to be resuspended in the surface water. As such, the fines content and water content of the FFT used in this experiment are considered to be generally representative of the FFT that may contribute to turbidity in BML. BML surface water was collected from the top 2 m of BML.

#### 2.1.2. Water Chemistry

Prior to biofilm growth and mixing experiments, the BML surface water and FFT pore water were characterized by measuring pH, electrical conductivity (EC), dissolved oxygen (DO), major cations and anions, naphthenic acids (NAs), and turbidity (in surface water only). pH and EC were measured using a Fisher Scientific Accumet AR50 Dual Channel pH/Ion/Conductivity Meter. DO was evaluated using a YSI 52 Dissolved Oxygen Meter (YSI Incorporated, Yellowsprings, OH, USA). Major cation analysis was conducted at the Natural Resources Analytical Laboratory (NRAL) at the University of Alberta. Samples were filtered with 0.45 µm nylon filters and analyzed for Na^+^, K^+^, Ca^2+^, and Mg^2+^ via inductively coupled plasma optical emission spectrometry (ICP-OES) (Thermo iCAP 600 series, Thermo Fisher Scientific, Cambridge, United Kingdom). Filtered samples (using 0.45 µm nylon filters) were analyzed for major anions (Cl^−^, SO_4_-S, NH_4_-N, NO_2_-N, NO_3_-N, and PO_4_-P) at NRAL with a Thermo Gallery Plus Beermaster Colorimetric Autoanalyzer (Thermo Fisher Scientific, Vantaa, Finland). NAs were determined using Fourier-transform infrared spectrometry (FTIR) (Spectrum 100 FT-IR Spectrometer, PerkinElmer, Shelton, CT, USA) following the procedure described in Ripmeester and Duford [31]. Turbidity was measured using a HACH 2100Q Portable Turbidimeter (Loveland, CO, USA).

#### 2.1.3. FFT Characterization

In addition to evaluating FFT pore water chemistry (see Section 2.1.2), the initial characterization of BML FFT (prior to biofilm growth and mixing tests) included measurements of solids and water content, bulk density, and particle size distribution. Sedimentation analysis was used to measure the particle size distribution and was conducted in accordance with ASTM International D79828-17: Standard Test Method for Particle-size Distribution (Gradation) of Fine-grained Soils Using the Sedimentation (Hydrometer) Analysis [32] using a 152H hydrometer (Fisherbrand, Buena, NJ, USA).

### 2.2. Biofilm Growth and Characterization

#### 2.2.1. Biofilm Growth

Mixing experiments were conducted in triplicate in 1-L pre-autoclaved glass jars, each containing 200 ± 1 g of FFT capped with 450 mL of BML surface water. BML surface water was carefully added to the jars to minimize the disturbance and suspension of FFT in the surface water. A total of 30 1-L jars were set up, and biofilms were grown in 18 of these jars. Prior to this experiment, naturally occurring biofilms were observed growing in two 1-L jars, each containing approximately 200 g of FFT and 450 mL BML water that had been stored at room temperature in natural light conditions for upwards of two years. Small samples (<0.5 mL) of these biofilms made up of microbial communities indigenous to FFT were pipetted into 18 of the jars containing FFT and BML surface water to enhance and accelerate biofilm growth for this experiment. Any FFT that was suspended as a result of the experimental setup was allowed to settle prior to the jar being inoculated with the biofilm. The remaining 12 control jars of FFT and BML surface water were sterilized by autoclaving (121 °C, 100 kPa) them three times. All 30 jars were then covered with a clear acrylic sheet to prevent evaporation of the surface water. The jars were stored at room temperature under a light intensity of approximately 60 µmol/m^2^/s, as suggested by Anderson and Kawachi [33], with a 16-h light: 8-h dark cycle to promote biofilm growth and to mimic the conditions under which the biofilms were initially grown. All jars were left under quiescent conditions for a 10-week or 20-week growth period before the mixing tests.

##### Biofilm Age

Previous research by Droppo et al. [16] noted the importance of the age of biofilms in their ability to stabilize underlying sediment and observed that lifting due to gas release and decay in older biofilms resulted in weaker attachment of the biofilm to the sediment. Thus, it was hypothesized that the age of biofilms may influence their ability to mitigate turbidity. To test this hypothesis, two different biofilm ages were examined: biofilms were grown for 20 weeks in 12 of the jars (hereinafter referred to as Biofilm20) and for 10 weeks in six of the jars (hereinafter referred to as Biofilm10) before conducting mixing tests. Control jars were placed under the same 20-week growth conditions as the Biofilm20 jars before conducting mixing tests. Based on laboratory observations, the 10-week and 20-week growth periods were selected because both periods were sufficient to grow biofilms that coated the surface of the FFT in the jars, but gas bubble release and subsequent lifting and layering of the biofilms were only evident under the 20-week growth period.

#### 2.2.2. Biofilm Characterization

Following biofilm development but before mixing tests, the BML surface water and FFT in the control jars and biofilm jars were characterized. The surface water was once again characterized by measuring pH, EC, DO, major cations and anions, initial turbidity, as well as total organic carbon (TOC) and chemical oxygen demand (COD). TOC was measured using a Shimadzu TOC-LCPH analyzer (Kyoto, Japan) (sparging time: 6 min; injection volume: 50 µL; injection number: 3 out of 4; acid added: 2.3%). COD was analyzed using HACH COD Digestion Vials (HR 20-1500 mg/L) in conjunction with a HACH Digital Reactor Box (DRB) 200 and a HACH DR 900 Multiparameter Portable Colorimeter (Loveland, CO, USA). FFT was characterized by measuring solids and water content and bulk density.

Prior to the mixing tests, the biofilms were characterized by measuring chlorophyll a (Chl *a*) and conducting LIVE/DEAD staining, scanning electron microscopy (SEM), and DNA extractions and 16S and 18S sequencing. Chl *a* was extracted using a method adapted from Thompson et al. [34]. Briefly, a sterile scalpel was used to cut approximately 1 cm^2^ biofilm samples, which were then placed in 2 mL of >99.9% methanol in the dark for 24 h at room temperature. The optical density (OD) of the chlorophyll/methanol mixture was measured at 665 and 652 nm using a Thermo Scientific NanoDrop 2000c Spectrophotometer (Wilmington, DE, USA). The Chl *a* content of the biofilms was then determined using Equation (1), taken from Lichtenthaler [35].

(1) $$\mathrm{Chl}\;a\;\left(\frac{\mathrm{mg}}{\mathrm{mL}\;\mathrm{methanol}}\right)=16.72*{\mathrm{OD}}_{665}-9.16*{\mathrm{OD}}_{652}$$

The viability of microorganisms in the biofilms was assessed using a LIVE/DEAD^®^ BacLight^TM^ Bacterial Viability Kit (L7012-Invitrogen, Carlsbad, CA, USA). A mixture of SYTO^®^ 9 green fluorescent nucleic acid stain and propidium iodide red fluorescent nucleic acid stain is used in this kit. A sterile scalpel was used to cut a thin section of the biofilm, which was then washed in phosphate-buffered saline (PBS) to remove any unattached biomass. Immediately after staining with the LIVE/DEAD^®^ BacLight^TM^ Bacterial Viability Kit, biofilm samples were incubated for 30 min in the dark. The stain was formulated from 3 µL of SYTO9 and propidium iodide dye mixture per mL. The distribution of live and dead cells could be observed via fluorescence; the fluorescent dye on live cells produced green fluorescence, while dead cells emitted bright red fluorescence. Prior to examining the biofilm samples under a microscope, the samples were washed with 0.85% sodium chloride (NaCl) to remove dye residues. A Zeiss Axio Imager M2 microscope (Jena, Germany) was used to analyze the stained biofilm samples with four FITC (red) and Cy3 (green) tracks. Image quantification was carried out using Fuji Image J software.

SEM was conducted on biofilm samples at the Microscopy Facility at the University of Alberta using a Zeiss EVO 10 Scanning Electron Microscope (Jena, Germany). In preparation for SEM analysis, biofilm samples were processed using a hexamethyldisilazane (HMDS) procedure. Briefly, the samples were placed in a fixative (2.5% glutaraldehyde; 2% paraformaldehyde in 0.1 M phosphate buffer), washed with a 0.1 M phosphate buffer, and dehydrated with a series of ethanol washes.

DNA extractions were conducted on biofilm samples and the initial FFT using a FastDNA^TM^ Spin Kit for Soils (MP Biomedical, Solon, OH, USA). DNA samples were sent to Microbiome Insights (Vancouver, BC, Canada) to perform polymerase chain reaction (PCR) amplification of the V4 region of the 16S rRNA gene in bacteria and archaea using the primers 515F (GTGCCAGCMGCCGCGGTAA) and 806R (GGACTACHVGGGTWTCTAAT) [36,37] and the V4 region of the 18S rRNA gene in eukaryotes using the primers 565F (CCAGCASCYGCGGTAATTCC) and 948R (ACTTTCGTTCTTGATYRA). Amplicon sequencing was conducted using the Illumina PE250 platform (Illumina, CA, USA). 16S sequencing was conducted on both biofilm samples and the initial BML FFT, while 18S sequencing was conducted on only the biofilm samples. The generated raw data were processed using the QIIME 2 (release 2018.8) next-generation microbiome bioinformatics platform [38]. The taxonomy was assigned with 99% similarity using the Greengenes 16S rRNA gene database (release gg_13_5) in accordance with McDonald et al. [39] and Werner et al. [40]. Sequencing reads were pre-processed, quality-filtered, and analyzed using the Divisive Amplicon Denoising Algorithm 2 (DADA2) software package, wrapped in QIIME version 2018.6.0. Statistical comparisons between the abundance values were performed using one-way ANOVA (Tukey’s test).

### 2.3. Mixing Tests

#### Mixing Methodology and Procedure

Following the 10-week and 20-week growth periods, mixing tests were conducted on the 1-liter jars to understand the influence of biofilms on turbidity mitigation in BML surface water. The mixing tests involved mechanical movement/mixing in the surface water and monitoring of subsequent turbidity generation over a period of 35 days. Lawrence et al. [11] estimated that the threshold linear velocity of FFT at the BML mudline is approximately 5 cm/s, meaning that the oscillating current at the mudline between the tailings and surface water must be greater than or equal to 5 cm/s to initiate erosion of the tailings. To simulate linear mudline velocities and generate turbidity in the 1-liter jars, a 48-millimeter diameter stainless steel blade impeller with a Caframo Constant Torque Mixer (BDC3030, Georgian Bluffs, Canada) was used. The offset distance between the mudline and impeller was set to 35 mm, equivalent to the midway point in the surface water. First, the threshold velocity of approximately 5 cm/s was confirmed in three control jars by mixing the water in the jars for 1 h at either 20, 30, or 40 rpm with the impeller stationary at a distance of 35 mm above the mudline (see Supplementary Table S1). These mixing speeds are equivalent to an average linear velocity of 2.5, 3.8, and 5.0 cm/s, respectively, using Equation (2) [41]:

(2) $$v=\frac{\omega *r}{2}$$

where *v* is the average linear velocity, ω is the angular velocity, and *r* is the impeller radius. After the threshold velocity was confirmed, the same test was conducted but jars were slowly moved in horizontal and vertical directions throughout the test. The purpose of this test was to confirm that the velocities along the mudline were comparable regardless of whether the impeller was stationary or moving. Because of the relatively small surface area of the mudline (65 cm^2^), turbidity was similar for both the stationary and moving impellers (see Supplementary Table S1). This test confirmed that a stationary impeller was not producing intense local velocities at the center of the mudline but was instead producing velocities all across the mudline (similar to that of a moving impeller).

Following these preliminary tests, each mixing test was conducted with a stationary impeller, mixing the BML surface water 35 mm above the mudline for 1 h. An initial mixing speed of 80 rpm was selected (equivalent to an average linear velocity of 10.1 cm/s) to evaluate the effectiveness of biofilms in mitigating turbidity generation. Following this test, higher mixing speeds of 120, 160, and 200 rpm were selected for the remaining jars to determine the mixing speed at which biofilms would ‘break’, generating turbidity in the jar. A summary of the different mixing speeds tested in triplicate during the mixing tests is provided in Table 1. Control jars (hereinafter referred to as No Biofilm jars) and Biofilm20 jars were tested in triplicate at each of the four mixing speeds. The six Biofilm10 jars were only tested at the two higher mixing speeds because it was hypothesized that these jars would perform better than the Biofilm20 jars.

After the 1-h mixing period, the FFT in the jars was allowed to settle for 35 days, at which point all turbidity curves had plateaued (see Section 3.2). Turbidity was measured immediately after the 1-h mixing period and every one to four days thereafter. pH and DO measurements were taken immediately after the 1-h mixing period and approximately every 10 to 15 days thereafter. The above mixing procedure was repeated after the suspended particles had settled and the jars were again monitored for 35 days. This second mixing test was conducted to see if biofilms continued to show improvement over No Biofilms even after the biofilms were disturbed due to mixing/movement in the surface water.

## 3. Results and Discussion

### 3.1. Surface Water, FFT, and Biofilm Characterization

#### 3.1.1. Water Chemistry and FFT Characterization

Table 2 presents water chemistry data for the surface water in the No Biofilm, Biofilm10, and Biofilm20 jars. All the measurements in Table 2 were taken after 10- or 20-week growth periods but prior to the mixing tests. Biofilms are known to develop in extreme environments. For example, Frederick [42] transferred established wetland benthic biofilms to slurries containing OSPW and noted that the presence of acutely toxic NAs in OSPW did not have a significant detrimental effect on biofilm growth. Furthermore, Sim et al. [43] found that high salinities (15, 45, and 70 ppt) did not limit benthic microbial biomass development, and that biofilm development slightly improved at higher salinities, likely a result of reduced competition and predation. In this work, biofilms were able to grow in the presence of NAs and elevated salts, with EC ranging from 2.36 to 3.01 mS/cm in the surface water and FFT (see also Supplementary Table S2). pH, EC, and chloride (Cl^−^) concentrations were slightly higher in the Biofilm10 and Biofilm20 surface water in comparison to No Biofilm surface water, while cation concentrations were fairly consistent across all jars.

Measurements of DO and TOC were also higher in the Biofilm10 and Biofilm20 jars, which is expected given the presence of algal communities in the biofilms that generate DO through photosynthesis and TOC through cell metabolism (further details in Section 3.1.2) [44,45]. The higher pH in the Biofilm10 and Biofilm20 surface water in comparison to the No Biofilm surface water is consistent with algae in biofilms consuming carbon dioxide (CO_2_) during photosynthesis. Nutrients in the Biofilm10 and Biofilm20 surface water were largely depleted in comparison to that of the No Biofilm jars, with lower concentrations of nitrogen (as ammonium (NH_4_-N), nitrate (NO_3_-N), and nitrite (NO_2_-N)) and phosphorous (as phosphate (PO_4_-P)). It is likely that nutrient concentrations in the FFT in the Biofilm20 and Biofilm10 jars were also lower than that of the No Biofilm jars [46]; however, the FFT pore water chemistry after biofilm growth was not evaluated, as pore water sampling would have substantially disturbed and damaged the overlying biofilm. In addition to nitrogen and phosphorous, potassium (K^+^) may act as a nutrient to support algal growth [47], and sulfate (SO_4_^2−^) can also support biofilm development, particularly in the presence of sulfate-reducing bacteria (SRB), colorless sulfur bacteria, or purple sulfur bacteria [22]. Sulfur concentrations (as SO_4_-S) were approximately 20% lower in the Biofilm10 and Biofilm20 surface water compared to the surface water in the No Biofilm jars. A similar trend was expected in the FFT pore water of these jars, though, again, this was not sampled to preserve the biofilm for the mixing tests. This trend is consistent with the SRB present in the biofilms, which is further discussed in Section 3.1.2. The initial turbidity was highest in the No Biofilm jars, but all initial turbidity values were less than 9 NTU.

Sedimentation analysis of the FFT revealed that the vast majority (more than 70 wt%) of particles were clay-sized, having a diameter of less than 2 µm. The median diameter (D_50_) of the FFT particles was 1.08 ± 0.14 µm. Table 3 presents further FFT characterization data for the FFT used in this study. All the measurements in Table 3 were taken prior to the mixing tests. The original solids content of the FFT was approximately 32.6 ± 0.0 wt%, with a corresponding water content of 67.4 ± 0.0 wt% and a bulk density of 1.13 ± 0.09 g/mL. Even after the 10-week or 20-week growth periods, the solids and water contents and bulk densities of the FFT in the Biofilm10, Biofilm20, and No Biofilm jars did not change substantially, which indicates that over the course of this experiment, self-weight consolidation of the FFT in the jars was minimal.

#### 3.1.2. Biofilm Characterization

The biofilms in all 18 jars were seen to visibly coat the surface of the FFT in the jars, equivalent to a surface area of 65 cm^2^ (see also Supplementary Figure S1). The biofilms visibly extended past the FFT surface in all the Biofilm20 jars and some of the Biofilm10 jars, and it was suspected that the biofilms had developed distinct, stratified layers [42]. Biofilm20 jars had noticeable gas bubbles form underneath the top layer of the biofilms, which resulted in lifting and then layering once the gas bubbles were released (see Supplementary Figure S2). In contrast, Biofilm10 jars had no gas bubbles, and the biofilms coating the FFT surface were smooth with no overlapping layers.

Prior to mixing, the Chl *a* concentration of the biofilms was measured as an indication of algal activity, such as green algae, diatoms, and cyanobacteria, and the results are presented in Table 2 [16,48]. Biofilm10 jars had a 46% higher Chl *a* concentration of 57.7 ± 6.0 mg/g biofilm compared to the Biofilm20 jars, which had a Chl *a* concentration of 39.4 ± 5.5 mg/g biofilm. Furthermore, there were noticeable color differences between biofilms of different ages. Biofilm10 jars contained biofilms that were strongly green, whereas the Biofilm20 jars had biofilms that were darker in color and were a mixture of brown and green. The differences in pigmentation and Chl *a* concentration in the Biofilm10 and Biofilm20 jars may reflect decaying algal communities or species succession in Biofilm20 jars [49]. Spatial variability in the collected samples due to heterogeneity within each biofilm may also contribute to differences between the Biofilm10 and Biofilm20 Chl *a* concentrations, though this is less likely given the differences in pigmentation between the two types of biofilm. Chl *a* concentration was also measured in the No Biofilm jars as a control, with a concentration of 0.28 ± 0.23 mg/g tailings.

**Figure 1:**
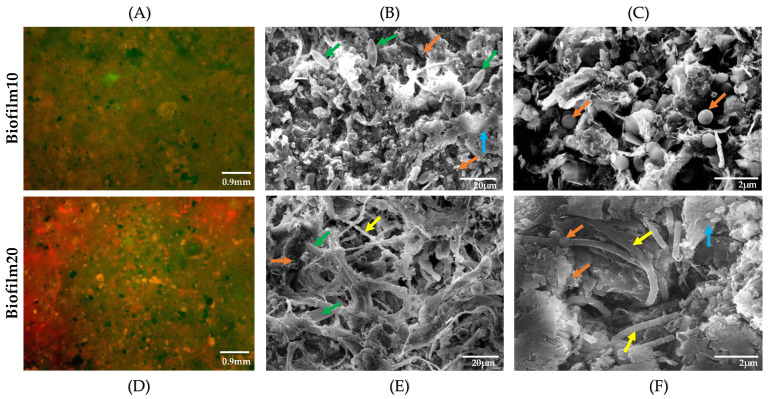
Viability of the cells was determined by LIVE/DEAD staining (**A**,**D**) at 200× magnification (green, live; red, dead). SEM was utilized to visualize the status of biofilms at week 10 (**B**,**C**) and week 20 (**E**,**F**). (**B**,**C**) are different SEM images of Biofilm10 taken at different scales—20 and 2 µm, respectively. Similarly, E and F are different SEM images of Biofilm20 taken at different scales—20 and 2 µm, respectively. Green arrows represent *Pinnularia* (phylum = Ochrophyta), yellow arrows represent filamentous cyanobacteria, orange arrows represent *Choricystis* (phylum = Chlorophyta), and blue arrows indicate EPS mixed with mineral particles. Biofilm10 corresponds to 10-week-old biofilms; Biofilm20 corresponds to 20-week-old biofilms. Note that only the top layer of biofilms was sampled for LIVE/DEAD staining and SEM imaging.

The Chl *a* concentrations are consistent with the DO, COD, and TOC water chemistry results in Table 2 and the LIVE/DEAD staining results in Figure 1. Prior to mixing, both Biofilm10 and Biofilm20 jars had high DO concentrations of 9.09 ± 0.44 and 8.18 ± 0.29 mg/L, respectively, compared to the No Biofilm jars, which had an average DO concentration of 5.05 ± 0.96 mg/L. The Biofilm20 jars had 11% lower DO on average than the Biofilm10 jars and had COD and TOC concentrations that were 11% and 12% higher on average, respectively, in comparison to that of the Biofilm10 jars. These results are consistent with the trend noted by He et al. [44], in which COD and TOC concentrations were highest during the stage of algae death and decay, likely due to a rapid release of intracellular organic matter into the surface water. Based on LIVE/DEAD staining of Biofilm10 (Figure 1A) and Biofilm20 (Figure 1D), it appears that Biofilm20 contained more dead cells than Biofilm10, though the biofilm matrix impacted the quality of these images. Taken together, these results indicate that decaying algal communities were likely more prevalent in the 20-week-old biofilms. However, only the top layer of biofilm was sampled for LIVE/DEAD staining in order to disturb the biofilms as little as possible. Because biofilm growth and development is a successional process, new layers of biofilm were likely integrating into FFT underneath the older top layer [16].

The biofilm topography under SEM showed a mixed appearance of bacteria and algae in both Biofilm10 and Biofilm20. Note that only the top layers of biofilms were sampled for SEM imaging. In Biofilm10 (Figure 1B,C), more species were visible; *Pinnularia* (diatoms) and coccoid *Choricystis* were witnessed profusely at 20 µm (Figure 1B), and a large colony of coccoid *Choricystis* appeared in Biofilm10 at a higher magnification of 2 µm (Figure 1C). The presence of diatoms (which belong to Ochrophyta) and *Choricystis* (which belong to Chlorophyta) in Biofilm10 is consistent with the 16S and 18S DNA sequencing results (discussed below). In contrast, observations of Biofilm20 revealed cyanobacteria, primarily of the filamentous type, and diatoms, most of which were coated in EPS at 20 µm (Figure 1E). At a higher magnification of 2 µm, clusters of coccoid *Choricystis* and filamentous cyanobacteria were visible coated in EPS (Figure 1F). The predominance of cyanobacteria in Biofilm20 and the presence of diatoms (which belong to Ochrophyta) and *Choricystis* (which belong to Chlorophyta) are also consistent with the 16S DNA sequencing results, which will be discussed further below. The higher amount of EPS evident in the SEM images of Biofilm20, in comparison to that of Biofilm10, is consistent with the older age of the biofilm [50].

Samples for DNA extraction and 16S and 18S sequencing were taken from the top layers of the biofilms in Biofilm20 and Biofilm10 before commencing the mixing tests. These top layer samples are referred to as Biofilm20a and Biofilm10a, respectively, in the following discussion. Distinct black and pink microbial communities (see Supplementary Figure S1) were visible below the FFT–water interface in the majority of the biofilm jars, and additional samples were taken from the Biofilm20 and Biofilm10 jars to capture these communities. These communities were especially prominent in the Biofilm20 jars, though they were evident to a lesser extent in many of the Biofilm10 jars. These additional samples are referred to as Biofilm10b and Biofilm10c and Biofilm20b and Biofilm20c in the following discussion (b: samples of pink microbial communities; c: samples of black microbial communities). The 18S sequencing data are only shown for Biofilm20a and Biofilm10a, as eukaryotes in the other biofilm samples were largely negligible.

**Figure 2:**
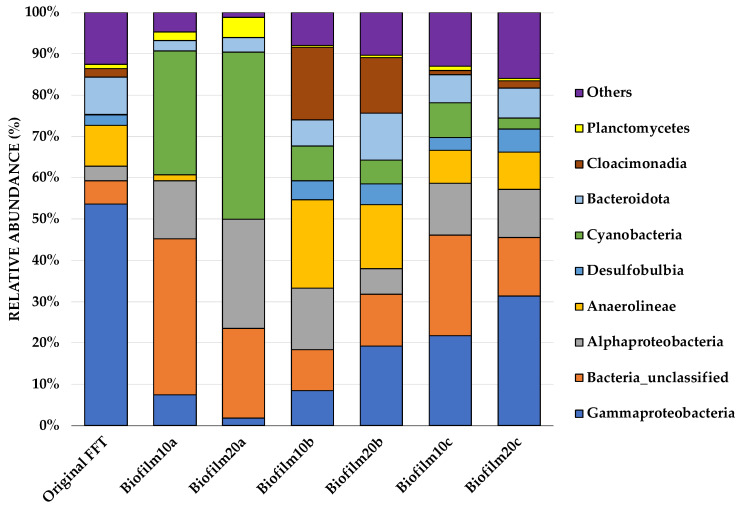
Stacking bars illustrating the relative abundances of microorganisms at the class level, represented by the percentage of the 16S rRNA read counts within each group. A relative abundance of less than 1% is assigned to ‘Others’. Original FFT refers to the FFT sampled before biofilms were inoculated and grown in jars. Biofilm10 corresponds to 10-week-old biofilms; Biofilm20 corresponds to 20-week-old biofilms. Biofilm10a and Biofilm20a refer to biofilm samples taken from the top layer of the biofilms. Biofilm10b and Biofilm20b refer to distinct pink microbial communities that were seen developing below the FFT–water interface (see Supplementary Figure S1). Biofilm10c and Biofilm20c refer to distinct black microbial communities that were seen developing below the FFT–water interface (see Supplementary Figure S1).

A predominance of Proteobacteria was observed at the phylum level, covering 33% of the overall microbial community, while other dominating phyla included Cyanobacteria (18.7%), Bacteria_unclassified (18.6%), Chloroflexi (8%), Desulfobacterota (7.2%) Bacteroidota (5.6%), Cloacimonadota (3.3%), and Planctomycetota (1.9%). All biofilm samples showed a complex and diverse microbial community to be more prominent at the class level. Figure 2 shows the 10 most abundant genera from each sample, augmented by many unclassified genera at the class level.

Sequences from the original FFT showed a predominance of *Gammaproteobacteria* at the class level, which represented 54% of the total community on average. Furthermore, *Gammaproteobacteria* were dominant in sample groups such as Biofilm20c (31.4%), Biofilm10c (21.8%), and Biofilm20b (19.3%); however, they were dramatically reduced in the top biofilm layers, Biofilm10a (7.4%) and Biofilm20a (1.8%). Compared to biofilm samples a and c, Biofilm10b had a relatively balanced, mixed community and consisted of only 8.5% *Gammaproteobacteria.* In aerobic primary cultures derived from froth treatment tailings, *Gammaproteobacteria* and other taxa are key players in degrading hydrocarbons [51]. Furthermore, *Alphaproteobacteria* and *Gammaproteobacteria* are the two most well-known groups of methanotrophic aerobic bacteria [52,53]. In comparison with the original FFT samples (3.6% on average), the *Alphaproteobacteria* population in all biofilms had doubled. Biofilm10a, Biofilm20a, and Biofilm10b contained the highest relative abundances of *Alphaproteobacteria* (14.1%, 26.3%, and 14.9%, respectively).

The phylum Chloroflexi, which contains *Anaerolineae*, is a well-known thermophilic bacterial group that was found in the original FFT samples (10% on average). Despite being absent from Biofilm20a and weakened sharply in Biofilm10a, they constituted 21.5% of the total bacterial count in Biofilm10b and 15.5% in Biofilm20b. The presence of thermophilic bacteria within the microbial community is not surprising given that tailings are exposed to high temperatures (historically up to 60°C) during processing [54], and Chloroflexi *(Anaerolineae*) have previously been reported in Syncrude tailings ponds [55,56]. *Desulfobulbia* is part of Desulfobacterota, which contribute to sulfate reduction, iron oxidation, and aromatic hydrocarbon degradation [57]. In nearly all samples, *Desulfobulbia* was present at an abundance of up to 6%, except in Biofilm10a and Biofilm20a. This is consistent with the decreased sulfur concentrations in the surface water in Biofilm10 and Biofilm20 jars presented in Table 2. While biofilm samples b and c (pink and black microbial communities, respectively) were fairly similar at the class level, Biofilm10b and Biofilm20b had notably higher proportions of *Anaerolineae* and *Cloacimonadia* relative to Biofilm10c and Biofilm20c. A dominant number of cyanobacteria was observed predominantly in Biofilm10a (30%) and Biofilm20a (40.5%) samples. In all other biofilm samples, cyanobacteria were present at an abundance of less than 10%. A very small percentage of cyanobacteria was also observed in the original FFT (0.12%).

**Figure 3:**
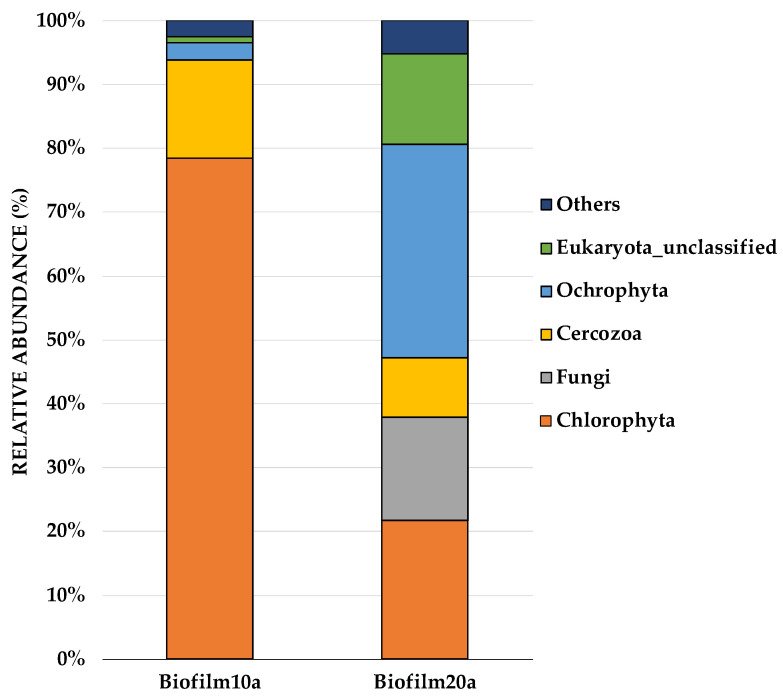
Stacking bars illustrating the relative abundances of Eukaryota at the phylum level in Biofilm10a and Biofilm20a, revealed by 18S sequencing. Microbial phyla that composed less than 1% of the dataset were grouped under the ‘Other’ category. Biofilm10 corresponds to 10-week-old biofilms; Biofilm20 corresponds to 20-week-old biofilms. Biofilm10a and Biofilm20a refer to biofilm samples taken from the top layer of the biofilms.

On the phylum-level distribution of whole eukaryotes, Chlorophyta (known as green algae) predominated (50%), followed by Ochrophyta (18%), Cercozoa (12%), Eukaryota_unclassified (8%), and fungi (8%). Figure 3 shows the most abundant Eukaryota from each sample, augmented at the phylum level. Interestingly, the most notable differences between biofilms of different ages (Biofilm10 and Biofilm20) occurred amongst the eukaryotes. The most dominant species in Biofilm10a were *Choricystis* sp. (observed in abundance under SEM), which belong to Chlorophyta, and *Cryptofilida_unclassified* sp., which belonged to Cercozoa. Biofilm20a was dominated by *Pinnularia brebissonii* and *Scenedesmus obliquus*, belonging to the phyla Ochrophyta and Chlorophyta, respectively (see Supplementary Figure S3). There was a notable difference among the Eukaryota phylum Chlorophyta, which accounted for 77% of Biofilm10a, compared to 22% of Biofilm20a. While Biofilm10a had a 55% higher relative abundance of Chlorophyta, Biofilm20a had a 10.5% higher relative abundance of cyanobacteria. Lifting and layering of Biofilm20 and/or algal self-shading may have limited light penetration, contributing to a greater proportion of cyanobacteria, which have higher growth rates than green algae under low light intensities, and a lesser amount of green algae in Biofilm20a [58].

In general, Biofilm10a and Biofilm20a samples contained higher proportions of aerobic photoautotrophic cyanobacteria and green algae, which is consistent with the top layer of the biofilms being exposed to light [42,59]. Samples taken from beneath the top layer (Biofilm10b and 10c and Biofilm20b and 20c) included higher proportions of facultative or anaerobic heterotrophs, such as *Gammaproteobacteria, Desulfobulbia,* and *Anaerolineae,* which may use organic carbon produced by the photoautotrophs as a carbon source [22,42,59]. The coexistence of heterotrophic bacteria and microalgae in biofilms is believed to result in a mutually beneficial relationship in which both parties rely less on external nutrient sources, EPS yields increase, and biostabilization is further enhanced [20]. Future research is needed to determine the factors that lead to specific microbial community structure changes within biofilms at different times, which will allow for a clearer understanding of how specific microorganisms play a role in the biostabilization of FFT.

### 3.2. Mixing Tests

**Figure 4:**
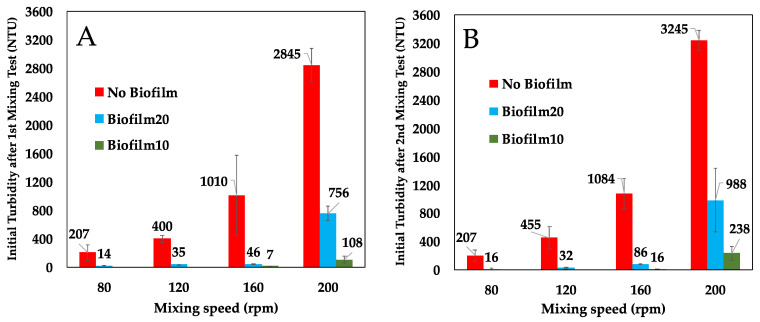
Comparison of initial turbidity in No Biofilm, Biofilm10, and Biofilm20 jars from the first and second mixing tests after 1-h mixing periods. (**A**) corresponds to the initial turbidity during the first mixing test, which commenced on Day 0; (**B**) corresponds to the initial turbidity during the second mixing test that commenced on Day 35. Results are presented as average ± one standard deviation of triplicates. Biofilm10 corresponds to 10-week-old biofilms; Biofilm20 corresponds to 20-week-old biofilms.

Figure 4 presents a comparison of the initial turbidity in the BML surface water in the No Biofilm, Biofilm10, and Biofilm20 jars immediately after the first and second 1-h mixing periods (which occurred 35 days apart). As anticipated, turbidity increased with mixing speed in all jars. However, the presence of a biofilm substantially reduced turbidity at all four mixing speeds during both tests. During the first mixing test, Biofilm20 reduced the initial turbidity in the overlying water by 73% to 95% on average, depending on the mixing speed, while Biofilm10 reduced the initial turbidity by 99% and 96% on average for mixing speeds of 160 and 200 rpm, respectively. The mixing test was conducted a second time to determine if biofilms continued to show improvement over No Biofilms even after the biofilms were ‘disturbed’ due to mixing/movement in the overlying surface water. The effectiveness of biofilms in mitigating turbidity decreased slightly when particles were resuspended during the second mixing test. During the second test, Biofilm20 reduced the initial turbidity by 69% to 93% on average, and Biofilm10 reduced turbidity by 93% to 99% on average compared to No Biofilm.

Biofilm10 performed better than Biofilm20 at both the 160 and 200 rpm mixing speeds. Biofilm20 had weaker attachment to the underlying FFT as evidenced by the lifting and layering of the top layers of these biofilms, despite having a higher amount of EPS as indicated by SEM imaging. This is likely a result of gas bubble releases and the higher proportion of decaying microbial communities in Biofilm20. Droppo et al. [16] found that the release of gas bubbles acted as a focal point for initiating ripping and failure of biofilms. As such, it is not surprising that Biofilm20 generated higher turbidity than Biofilm10 [16,19]. Prior to the mixing tests, the top layer of the biofilms acted as sheets covering the FFT surface and isolating the underlying FFT from the surface water. During the first mixing test, at a mixing speed of 200 rpm in the Biofilm10 jars and Biofilm20 jars, the top layers of the biofilms rolled up or folded over onto themselves, exposing the underlying biostabilized FFT and leading to erosion of the FFT and higher turbidities in the surface water. Because of the weaker attachment of Biofilm20 to the underlying FFT, larger portions of these biofilms rolled up or folded over in comparison to Biofilm10. Furthermore, during both the first and second mixing tests, isolated failures (ripping) of Biofilm20 occurred at mixing speeds of 120, 160, and 200 rpm, again leading to higher turbidities. These isolated failures were not evident during the Biofilm10 mixing tests. Biofilm20 essentially remained intact at a mixing speed of 80 rpm, and Biofilm10 remained intact at a mixing speed of 160 rpm.

The consistently higher turbidity measured in Biofilm20 compared to Biofilm10 was predicted, based on the work of Droppo et al. [16]. Because biofilm growth and development is a successional process, biofilms are likely to experience fluctuations in their ability to stabilize sediment over time [16]. Processes such as microbial death and decay, species succession, and changes to EPS production and distribution are thought to have a significant effect on biofilm stability. As such, as biofilms age, there may be periods of increased stability and periods of decreased stability. Regardless, biostabilized sediment is expected to have improved stability over purely mineral sediments [16].

**Figure 5:**
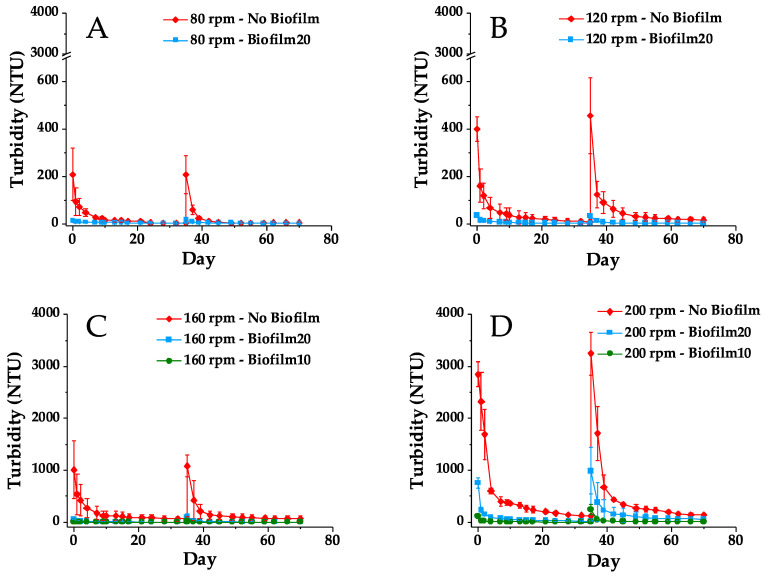
Turbidity in No Biofilm, Biofilm10, and Biofilm20 jars during first and second mixing tests. (**A**–**D**) correspond to mixing speeds of 80, 120, 160, and 200 rpm, respectively. Results are presented as average ± one standard deviation of triplicates. The spike in turbidity at Day 35 corresponds to the turbidity immediately after the second 1-h mixing period. Note that A and B have y-axis breaks between 600 and 3000 NTU. Biofilm10 corresponds to 10-week-old biofilms; Biofilm20 corresponds to 20-week-old biofilms.

Figure 5 presents turbidity in No Biofilm, Biofilm10, and Biofilm 20 jars measured over the course of the first and second mixing tests. The spikes in turbidity at Day 35 correspond to the turbidity immediately after the second 1-h mixing period. The No Biofilm jars had the highest turbidity for all four mixing speeds during both the first and second mixing tests, and they also showed the most variability in the triplicate turbidity measurements. This is likely a result of the high turbidity generated in these jars as well as variability in the FFT solids content and particle sizes. Biofilm20 jars also showed greater variability in triplicate turbidity measurements during the second 200 rpm mixing test, when turbidity measurements were highest.

At all mixing speeds, the turbidity in the Biofilm20 and Biofilm10 jars dissipated faster than that of the No Biofilm jars. This is a result of both the lower turbidity generated in the biofilm jars and the size of the flocs being suspended. While FFT was suspended in both No Biofilm and biofilm jars, visibly larger flocs were suspended in the biofilm jars. These flocs were presumably biostabilized FFT and pieces of the biofilm that had ripped off from the rest of the biofilm coating the FFT surface [19]. This observation is consistent with the results of Droppo et al. [15], who noted that due to the strong binding nature of biofilms, the erosion of biostabilized sediment generated larger and more stable flocs relative to purely mineral sediment.

As illustrated in Figure 4 and Figure 5, the turbidity was higher during the second mixing test compared to the first mixing test for all jars and all mixing speeds, with the exception of Biofilm20 jars mixed at 120 rpm, which saw an initial turbidity decrease of 3 NTU on average, and No Biofilm jars mixed at 80 rpm, which saw no change in turbidity between the first and second mixing tests. When comparing the percent difference in initial turbidity measured after the first and second 1-h mixing periods (see Supplementary Figure S4), biofilm jars showed the greatest percent increase in turbidity. For example, during the second 1-h mixing period, the initial turbidity in Biofilm10 jars mixed at 160 or 200 rpm more than doubled compared to that of the first mixing period 35 days prior. Despite this increase in turbidity, the presence of biofilms still showed improvement over No Biofilms during the second mixing test, even though most of the biofilms were disturbed to some extent during the first mixing test, either by ripping apart, rolling up, and/or folding over.

The higher turbidity in the biofilm jars during the second mixing test is likely due to a combination of factors, including increasing biofilm age and thereby death and decay of the older uppermost layers of the biofilms, reduced biofilm integrity as a result of mechanical mixing, and decreased photosynthesis and algal growth due to turbidity. The mixing tests appeared to impact algal growth in the biofilms as evidenced by changes in the DO concentrations in the surface water immediately following the 1-h mixing periods (see Supplementary Figure S5). Immediately after both the first and second 1-h mixing periods, the DO in the Biofilm20 and Biofilm 10 jars decreased, especially in the Biofilm20 jars mixed at higher speeds. Conversely, the No Biofilm jars experienced a spike in DO immediately after the first and second 1-h mixing periods due to aeration from mechanical mixing. The DO in the biofilm jars continued to decrease for at least 10 days following the two 1-h mixing periods before gradually recovering. Thus, photosynthesis was likely temporarily hindered by the turbidity in the biofilm jars. This agrees with previous studies, which have found that turbidity and light penetration are dominant factors limiting algal biofilm production [42,60,61,62]. The decrease in DO in the biofilm jars may also have been due to an increase in oxygen demand following the mixing periods, though oxygen demand was not measured during the mixing tests. Turbidity generation in the biofilm jars (due to the suspension of biostabilized FFT and pieces of biofilms) may have increased the amount of TOC and heterotrophs in the surface water, both of which could increase the oxygen demand and thus contribute to the decrease in DO in the biofilm jars. At the end of the two 35-day mixing tests (Day 70), the DO concentrations in all of the No Biofilm jars were greater than or equal to their initial (Day 0) concentration of 5.05 ± 0.96 mg/L. However, the DO concentrations in the Biofilm20 jars on Day 70 were 1.36 to 1.99 mg/L lower than their Day 0 concentration of 8.18 ± 0.29 mg/L, depending on the mixing speed. The higher mixing speeds in the Biofilm20 jars corresponded with lower Day 70 DO concentrations. The DO concentrations in all six Biofilm10 jars on Day 70 were approximately 1.9 mg/L lower than their Day 0 concentration of 9.09 ± 0.44 mg/L, regardless of mixing speed. The decrease in DO in the biofilm jars over the course of the first and second mixing tests is likely a result of turbidity inhibiting photosynthesis and increasing oxygen demand, as well as microbial death and decay and species succession. The pH of the surface water remained relatively stable in all jars throughout the mixing tests (see Supplementary Figure S6).

The No Biofilm jars mixed at 120, 160, and 200 rpm also had a turbidity increase during the second mixing test. This may be partly because the turbidity in the No Biofilm jars mixed at 160 and 200 rpm was still relatively high, at 62 and 115 NTU, respectively, just prior to commencing the second mixing test compared to that of the Biofilm10 and Biofilm20 jars. Additionally, recently settled FFT particles in the No Biofilm jars could have created an intermediate layer that was less dense and more mobile than the underlying FFT [11]. This material would be more easily and quickly resuspended during the second mixing test, and the resuspension of this intermediate layer would lead to the exposure and subsequent erosion of the underlying FFT. The development of this intermediate layer is most likely to have occurred in the No Biofilm jars mixed at higher speeds (160 and 200 rpm) because of the greater amount of FFT particles that were suspended at these mixing speeds.

Though consolidation has been found to contribute to the stabilization of bed sediments [23,24], this effect is expected to have been minimal in this experiment because of the more significant role of biostabilization [19] and because of the fine-grained nature of the FFT and the small amount of tailings (approximately 200 g) in each jar. After the 10-week or 20-week growth period, the solids and water contents and bulk densities of the FFT in the Biofilm10, Biofilm20, and No Biofilm jars did not change substantially from those of the initial FFT, which indicates that self-weight consolidation of the FFT in the jars was minimal, and therefore, consolidation did not contribute to stabilization. Electrochemical interactions in the FFT may have generated some attraction and stabilizing forces [19]. However, because of the substantial turbidity mitigation observed in the biofilm jars compared to the No Biofilm jars, it can be concluded that biostabilization was the dominant factor contributing to reduced FFT turbidity in this work. While microbial communities in the biofilms could have impacted FFT geochemistry, potentially influencing interparticle binding and increasing or decreasing stability [16], the secretion of EPS is still thought to be the primary mechanism by which biofilms enhance stabilization [14,19,25,26,27].

In this work, mudline biofilms made up of diverse communities of photoautotrophs and heterotrophs indigenous to oil sands tailings substantially reduced turbidity generated as a result of mixing/movement in the overlying surface water. However, the growth of biofilms on the BML mudline and subsequent biostabilization likely depend on a number of factors, including temporal seasonal changes [19,26] and the depth of light penetration in the surface water. Predominately algal biofilms may develop in shallow locations within BML, similar to the algal biofilms that naturally occur in surrounding wetlands [42], while bacterial biofilms may dominate at greater depths or in particularly turbid locations. Additionally, further investigation is necessary to determine how gas ebullition and pore water expression from FFT into overlying surface water may impact the growth and structure of biofilms and turbidity mitigation. The biofilms in this experiment were grown under quiescent conditions, which is also likely to impact biofilm growth and structure, and as such, the impact of surface water mixing/movement during biofilm growth should also be investigated in future studies.

The mixing methodology used in this experiment was not intended to replicate field conditions, but rather to evaluate biofilms as a potential turbidity mitigation mechanism. Turbidity generation in BML due to mixing/movement in the surface water is a result of a number of processes, as previously discussed, including wind waves and other wind-driven processes and convection [11]. These processes may occur in short bursts or intermittently over several days, such as during lake turnover, and the extent to which these processes generate turbidity will depend on several factors, including the depth of the surface water cap. The mixing methodology used in this study involved short (1 h) mixing periods at increasing speeds and linear velocities in order to first evaluate the effectiveness of biofilms in mitigating turbidity generation and second to determine the mixing speed at which biofilms were disturbed, either by ripping, rolling up, and/or folding over, and subsequently generated turbidity in the surface water. The highest mixing speeds tested in this study do not necessarily reflect scenarios under which turbidity will be generated in BML but rather demonstrate the high mixing speed required to disturb otherwise intact biofilms. As such, while the mixing duration and mudline linear velocities used in this work do not reflect all scenarios under which turbidity may be generated in BML, the results clearly show that the presence of biofilms at the mudline reduced turbidity generation under all mixing conditions evaluated in this work.

## 4. Conclusions

This study is the first to investigate and demonstrate that biostabilization is a promising mechanism for turbidity mitigation in EPLs. Mudline biofilms made up of photoautotrophs and heterotrophs indigenous to oil sands tailings reduced surface water turbidity by 69% to 99% during laboratory mixing experiments. The 20-week-old biofilms were more prone to lifting and layering due to gas release and microbial death and decay in comparison to 10-week-old biofilms, which led to weaker attachment of older biofilms to underlying FFT and higher turbidity measurements. However, regardless of biofilm age, the presence of biofilms substantially reduced surface water turbidity in comparison to sterilized FFT.

## Figures and Tables

**Table 1:** Summary of mixing speeds and biofilm ages examined in mixing experiments. Each test was conducted in triplicate. Biofilm10 corresponds to 10-week-old biofilms; Biofilm20 corresponds to 20-week-old biofilms.

	Mixing Speed (rpm)			
	80	120	160	200
No Biofilm	X	X	X	X
Biofilm10			X	X
Biofilm20	X	X	X	X

**Table 2:** Surface water chemistry data and Chl *a* biofilm concentrations in No Biofilm, Biofilm10, and Biofilm20 jars prior to mixing tests. Results are presented as average ± one standard deviation of replicates. Biofilm10 corresponds to 10-week-old biofilms; Biofilm20 corresponds to 20-week-old biofilms.

Parameter	No Biofilm	Biofilm10	Biofilm20
pH	8.78 ± 0.08	9.08 ± 0.02	9.06 ± 0.04
EC (mS/cm)	2.69 ± 0.05	2.92 ± 0.16	3.01 ± 0.23
DO (mg/L)	5.05 ± 0.96	9.09 ± 0.44	8.18 ± 0.29
TOC (mg/L)	59.3 ± 4.2	70.2 ± 1.9	78.5 ± 3.2
COD (mg/L)	280.7 ± 6.7	281.3 ± 5.8	313.0 ± 10.0
NH_4_-N (mg/L)	3.84 ± 0.13	0.030 ± 0.014	0.026 ± 0.007
NO_2_-N (µg/L)	7.46 ± 2.42	3.15 ± 1.47	1.22 ± 1.01
NO_3_-N (mg/L)	0.045 ± 0.007	0.050 ± 0.007	0.018 ± 0.008
PO_4_-P (µg/L)	13.4 ± 2.9	7.46 ± 1.44	7.08 ± 1.61
SO_4_-S (mg/L)	99.9 ± 3.6	81.6 ± 12.9	78.3 ± 12.7
Cl^−^ (mg/L)	381.4 ± 8.7	414.1 ± 18.3	423.8 ± 52.6
Na^+^ (mg/L)	608.3 ± 10.9	611.9 ± 18.4	610.6 ± 67.1
K^+^ (mg/L)	15.3 ± 0.6	13.7 ± 1.0	14.7 ± 2.0
Ca^2+^ (mg/L)	9.6 ± 0.1	8.8 ± 0.2	8.8 ± 1.4
Mg^2+^ (mg/L)	5.4 ± 0.5	7.4 ± 0.3	6.8 ± 1.2
Turbidity (NTU)	8.93 ± 5.34	2.53 ± 0.75	4.67 ± 3.88
Chl *a* (mg/g biofilm)	0.28 ± 0.23	57.7 ± 6.0	39.4 ± 5.5

**Table 3:** FFT characterization data for the initial BML FFT and FFT in No Biofilm, Biofilm10, and Biofilm20 jars prior to mixing tests. Results are presented as average ± one standard deviation of replicates. Biofilm10 corresponds to 10-week-old biofilms; Biofilm20 corresponds to 20-week-old biofilms.

Parameter	Initial BML FFT	No Biofilm	Biofilm10	Biofilm20
Solids content (wt%)	32.6 ± 0.0	31.6 ± 0.7	32.5 ± 0.9	31.9 ± 0.6
Water content (wt%)	67.4 ± 0.0	68.4 ± 0.7	67.5 ± 0.9	68.1 ± 0.6
Bulk density (g/mL)	1.13 ± 0.09	1.12 ± 0.05	1.13 ± 0.11	1.13 ± 0.06

## Data Availability

All data generated or used during the study appear in the submitted manuscript and the Supplemental Data.

## Supplementary Materials

The following are available online at https://www.mdpi.com/article/10.3390/microorganisms9071443/s1, Table S1: Turbidity measurements from preliminary mixing tests; Table S2: Water chemistry data for initial BML surface water and initial FFT pore water; Figure S1: Picture of Biofilm10 and Biofilm20 jars; Figure S2: Picture of A: Gas bubble in a Biofilm 20 jar and B: Lifting and layering of Biofilm20 following gas bubble release; Figure S3: Comparison of Eukaryote abundance at the species level in Biofilm10a and Biofilm20a; Figure S4: Percent difference in average initial turbidity generated during the first and second 1-h mixing periods for No Biofilm, Biofilm10, and Biofilm20 jars; Figure S5: Dissolved oxygen (DO) concentrations in No Biofilm, Biofilm10, and Biofilm20 jars during the first and second mixing tests; Figure S6: pH in No Biofilm, Biofilm10, and Biofilm20 jars during the first and second mixing tests.
