# Supplementary material for: Biofilms for Turbidity Mitigation in Oil Sands End Pit Lakes
Source: Microorganisms. 2021 Jul 4;9(7):1443. doi: 10.3390/microorganisms9071443 (PMC8307862; doi:10.3390/microorganisms9071443)
Supplement: Supplementary file 1 [file microorganisms-09-01443-s001.zip › microorganisms-1260135 supplementary material.pdf]

Supplemental Data

This supplement includes turbidity data from preliminary mixing tests (Table S1) and water chemistry data for the initial BML surface water and initial FFT pore water used in this work (Table S2). Figure S1 shows pictures of Biofilm10 and Biofilm20 jars prior to mixing tests. Figure S2 shows pictures of a gas bubble in a Biofilm 20 jar, and the subsequent lifting and layering of Biofilm20 after the gas bubble was released. Figure S3 provides a comparison of Eukaryote abundance at the species level in Biofilm 10a and Biofilm 20a. Figure S4 compares the initial turbidity generated during the first and second 1-hour mixing periods using percent difference. DO and pH measurements taken over the course are of 70-day experiment are available in Figures S5 and S6, respectively.

Table S1: Turbidity measurements from preliminary mixing tests to confirm the threshold velocity of 40 rpm and compare turbidity generation from stationary versus moving impellers.

Mixing speed (rpm)	Turbidity after 1 hour (NTU)	
	Stationary Impeller	Moving Impeller
20	12.2	16.7
30	17.3	19.5
40	87.0	91.1

Table S2: Water chemistry data for initial BML surface water and initial FFT pore water prior to biofilm growth and mixing tests. Results are presented as average ± one standard deviation of triplicates.

Parameter	Initial BML Surface Water	FFT Pore Water
pH	8.52 ± 0.04	8.18 ± 0.03
EC (mS/cm)	2.36 ± 0.01	2.95 ± 0.01
DO (mg/L)	6.96 ± 0.11	1.96 ± 0.07 *
NH_4-N (mg/L)	0.329 ± 0.004	7.12 ± 0.17
NO_2-N (μg/L)	0.181 ± 0.032	12.8 ± 11.9
NO_3-N (mg/L)	0.461 ± 0.097	0.168 ± 0.029
PO_4-P (μg/L)	2.96 ± 0.11	112.6 ± 47.4
SO_4-S (mg/L)	60.4 ± 0.5	18.8 ± 1.7
Cl^- (mg/L)	334.4 ± 2.4	476.3 ± 5.1
Na^+ (mg/L)	546.0 ± 2.4	791.5 ± 7.4
K^+ (mg/L)	14.2 ± 0.1	21.0 ± 1.4
Ca^{2+} (mg/L)	23.5 ± 0.1	22.0 ± 2.1
Mg^{2+} (mg/L)	11.9 ± 0.1	12.9 ± 0.1
Naphthenic acids (mg/L)	45.3 ± 6.7	70.5 ± 11.7
Turbidity (NTU)	2.75 ± 0.96	-

*Measured in FFT (includes both solids and pore water)

SUPPLEMENTAL DATA

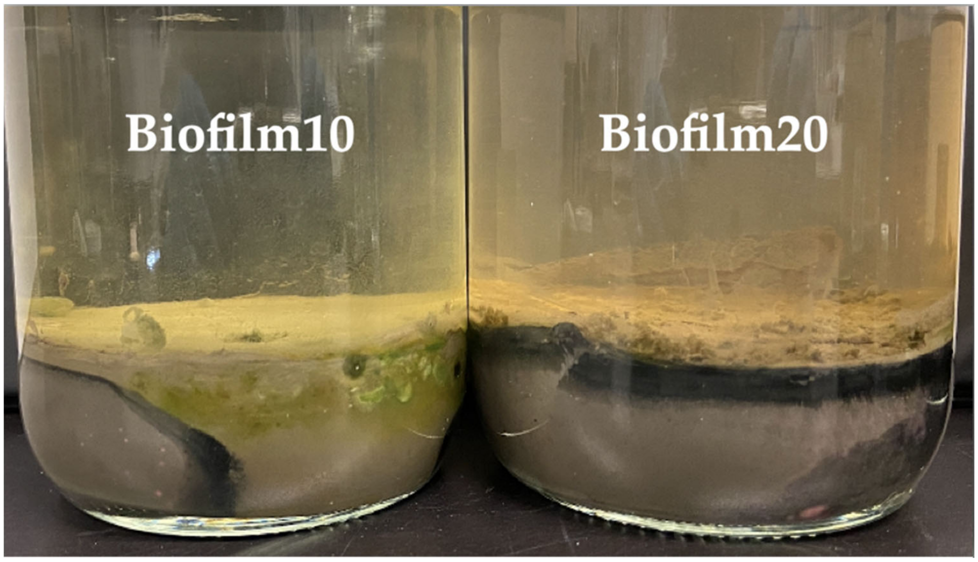

Figure S1: Picture of a Biofilm10 (left) jar and a Biofilm20 (right) jar after 10-week and 20-week growth periods, respectively, but prior to mixing tests. Biofilm10 corresponds to 10-week-old biofilms; Biofilm20 corresponds to 20-week-old biofilms.

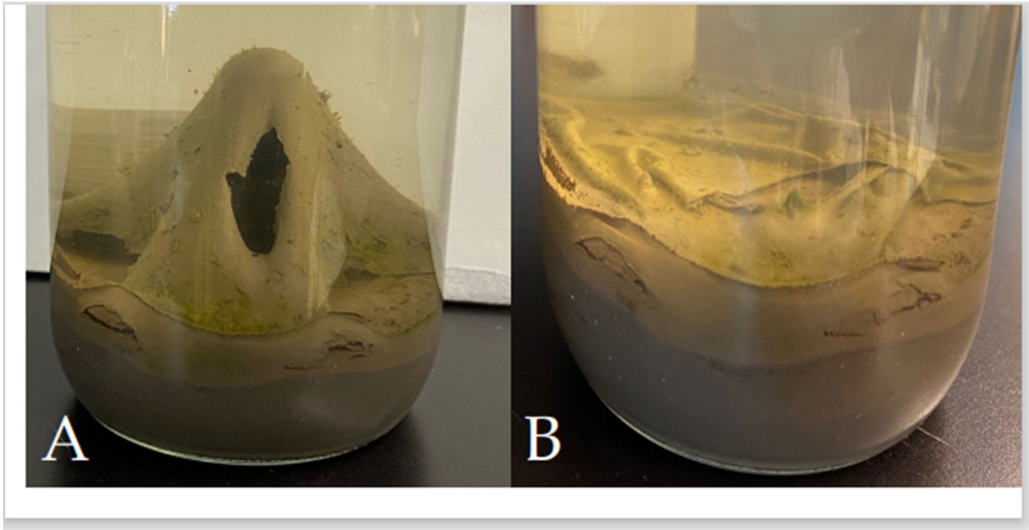

Figure S2: Picture of A: Gas bubble in a Biofilm20 jar and B: Subsequent lifting and layering of Biofilm20 after gas bubble release during 20-week growth period. Biofilm20 corresponds to 20-week-old biofilms.

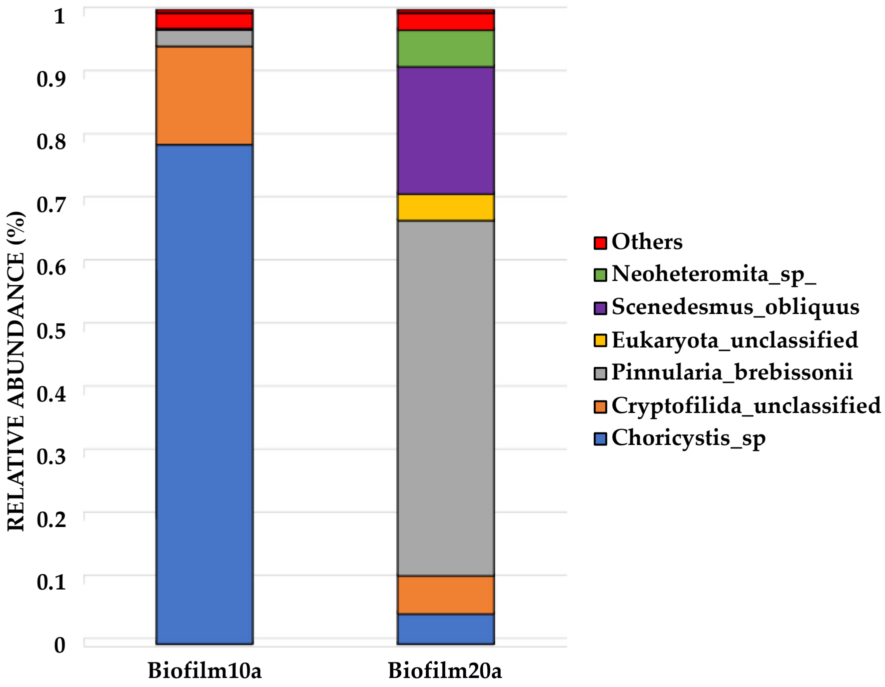

Figure S3: A comparison of Eukaryote abundance at the species level in Biofilm 10a and Biofilm 20a, revealed by 18S sequencing. Biofilm10 corresponds to 10-week-old biofilms; Biofilm20 corresponds to 20-week-old biofilms. Biofilm10a and Biofilm20a refer to biofilm samples taken from the top layers of the biofilms.

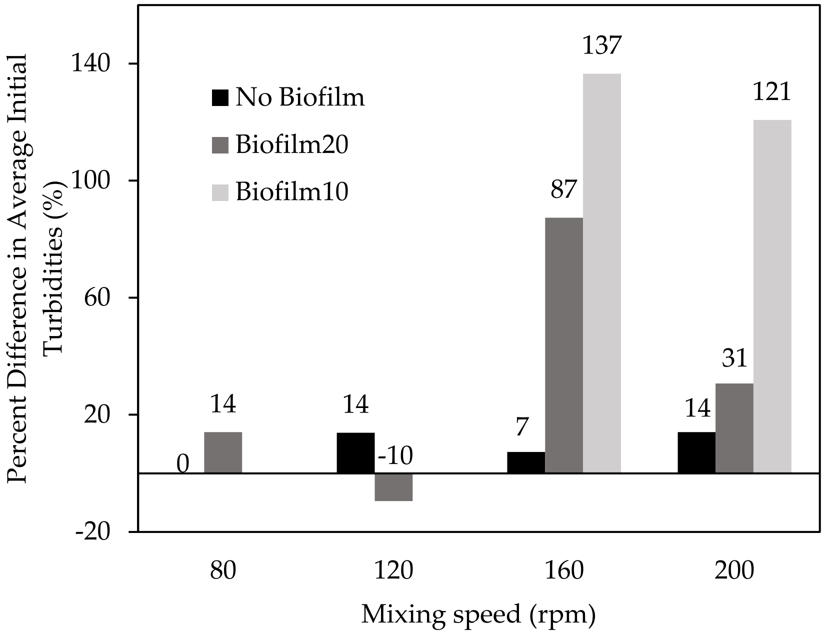

Figure S4: Percent difference in average initial turbidity generated during the first and second 1-hour mixing periods for No Biofilm, Biofilm10, and Biofilm20 jars. Biofilm10 corresponds to 10-week-old biofilms; Biofilm20 corresponds to 20-week-old biofilms.

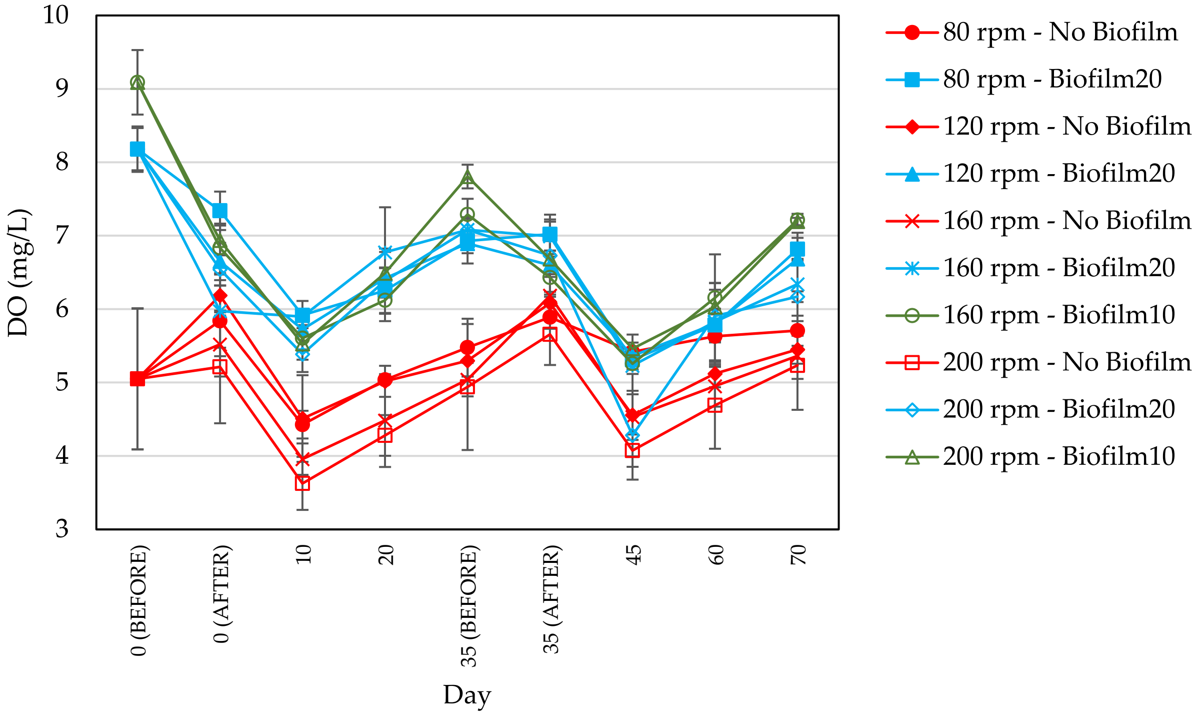

Figure S5: Dissolved oxygen (DO) concentrations in No Biofilm, Biofilm10, and Biofilm20 jars during the first and second mixing tests. Before and After labels in the x-axis indicated measurements taken immediately before or after the first or second 1-hour mixing period, which occurred on Days 1 and 35. Biofilm10 corresponds to 10-week-old biofilms; Biofilm20 corresponds to 20-week-old biofilms.

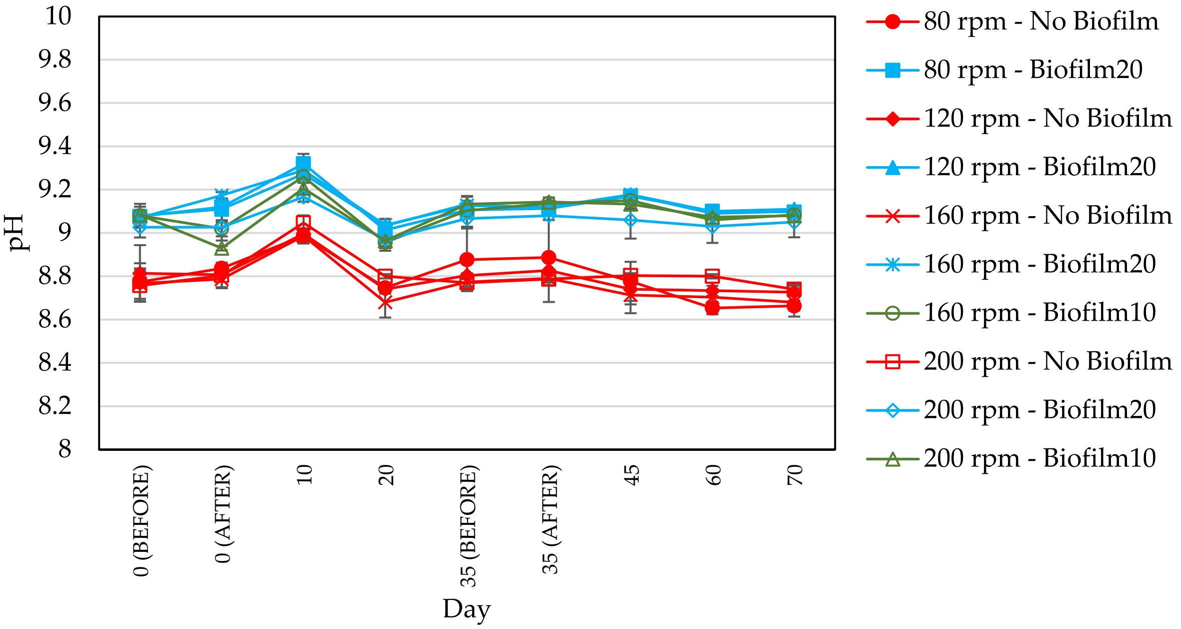

Figure S6: pH in No Biofilm, Biofilm10, and Biofilm20 jars during the first and second mixing tests. Before and After labels in the x-axis indicated measurements taken immediately before or after the first or second 1-hour mixing period, which occurred on Days 1 and 35. Biofilm10 corresponds to 10-week-old biofilms; Biofilm20 corresponds to 20-week-old biofilms.
